# Prioritizing Crop Management to Increase Nitrogen Use Efficiency in Australian Sugarcane Crops

**DOI:** 10.3389/fpls.2017.01504

**Published:** 2017-09-05

**Authors:** Peter J. Thorburn, Jody S. Biggs, Jeda Palmer, Elizabeth A. Meier, Kirsten Verburg, Danielle M. Skocaj

**Affiliations:** ^1^Queensland Bioscience Precinct, Commonwealth Scientific and Industrial Research Organisation, St Lucia QLD, Australia; ^2^Black Mountain Science and Innovation Precinct, Commonwealth Scientific and Industrial Research Organisation, Canberra ACT, Australia; ^3^Sugar Research Australia, Tully QLD, Australia

**Keywords:** Great Barrier Reef, fallow management, nitrogen fertilizer, modeling, APSIM

## Abstract

Sugarcane production relies on the application of large amounts of nitrogen (N) fertilizer. However, application of N in excess of crop needs can lead to loss of N to the environment, which can negatively impact ecosystems. This is of particular concern in Australia where the majority of sugarcane is grown within catchments that drain directly into the World Heritage listed Great Barrier Reef Marine Park. Multiple factors that impact crop yield and N inputs of sugarcane production systems can affect N use efficiency (NUE), yet the efficacy many of these factors have not been examined in detail. We undertook an extensive simulation analysis of NUE in Australian sugarcane production systems to investigate (1) the impacts of climate on factors determining NUE, (2) the range and drivers of NUE, and (3) regional variation in sugarcane N requirements. We found that the interactions between climate, soils, and management produced a wide range of simulated NUE, ranging from ∼0.3 Mg cane (kg N)^-1^, where yields were low (i.e., <50 Mg ha^-1^) and N inputs were high, to >5 Mg cane (kg N)^-1^ in plant crops where yields were high and N inputs low. Of the management practices simulated (N fertilizer rate, timing, and splitting; fallow management; tillage intensity; and in-field traffic management), the only practice that significantly influenced NUE in ratoon crops was N fertilizer application rate. N rate also influenced NUE in plant crops together with the management of the preceding fallow. In addition, there is regional variation in N fertilizer requirement that could make N fertilizer recommendations more specific. While our results show that complex interrelationships exist between climate, crop growth, N fertilizer rates and N losses to the environment, they highlight the priority that should be placed on optimizing N application rate and fallow management to improve NUE in Australian sugarcane production systems. New initiatives in seasonal climate forecasting, decisions support systems and enhanced efficiency fertilizers have potential for making N fertilizer management more site specific, an action that should facilitate increased NUE.

## Introduction

Crop production often relies on the use of nitrogen (N) fertilizer. However, the application of N fertilizer increases the risk of N being lost to the environment, either as greenhouse gases or carried in water to groundwater, aquatic, and/or marine ecosystems (Schlesinger, 2009). Impacts of loss of N from agricultural lands have been well documented in many regions (Burkart and James, 1999; Mitsch et al., 2001; Jalali, 2005; Howarth, 2008; National Research Council, 2008; Oenema et al., 2009). Environmental losses of N are stimulated by increased applications of fertilizer because not all N is taken up by the crop and removed from the field, or stored in the soil (especially in the long-term). Typically, around only 40% of N fertilizer is incorporated into harvested products that are exported from the field (Ladha et al., 2005), although there is considerable variability in this percentage. The remaining N may potentially be lost from the site (Schlesinger, 2009; Canfield et al., 2010). Given the concerns over the environmental impacts of N in agriculture, understanding the efficiency with which N fertilizer is incorporated into crops is an important topic. A simple and widely used measure of this efficiency is N use efficiency (NUE), which is the mass of harvested product relative to the mass of N applied to the field (Fageria and Baligar, 2005). Understanding NUE and identifying ways to increase it is a topic that has received substantial attention (Fageria and Baligar, 2005; Ladha et al., 2005; Dawson et al., 2008; Wezel et al., 2014).

Tropical areas have recently undergone extensive agricultural intensification (FAO and JRC, 2012), which has had flow on consequences for aquatic and/or marine ecosystems in many areas, for example, in Brazil (Filoso et al., 2003; Martinelli et al., 2010), Africa (Olago and Odada, 2007; van der Laan et al., 2012), and northern Australia (Brodie et al., 2013; Thorburn et al., 2013b; Kroon et al., 2016). Sugarcane (*Saccharum* spp.) is an important crop in tropical and sub-tropical areas, where it often makes a substantial contribution to the local economy (Moore et al., 2014). It is also important globally, as one of the largest sources of energy for human consumption (Moore et al., 2014) and a major source of biofuels (Müller-Langer et al., 2014). Sugarcane production relies heavily on the use of N fertilizer (FAO, 2006) because substantial amounts of N (Keating et al., 1999) are contained in the above-ground biomass of mature crops. The high application rates of N fertilizer increase the likelihood of environmental impacts of sugarcane production, particularly in regions close to environmentally sensitive areas. Indeed, N lost from sugarcane cropping systems is implicated in the impacts on tropical ecosystems noted above (Martinelli and Filoso, 2008; Martinelli et al., 2010; van der Laan et al., 2012). An important example is Australia (Thorburn et al., 2003a; Brodie et al., 2012, 2013; Kroon et al., 2016) where the majority of sugarcane is grown in environmentally sensitive areas; namely in catchments that drain directly into the Great Barrier Reef Lagoon, a world heritage listed ecosystem of great ecological and economic value. As well as aquatic ecosystem impacts, emissions of the greenhouse gas nitrous oxide during sugarcane production are a concern as they both contribute to global warming and reduce the energy yield of bioenergy produced from sugarcane (Hartemink, 2008; Lisboa et al., 2011), something that has spurred the search for improved NUE in sugarcane biofuel production (Otto et al., 2016). Thus there is a clear imperative to better understand and improve NUE in sugarcane cropping systems, especially those in Australia.

**FIGURE 1:**
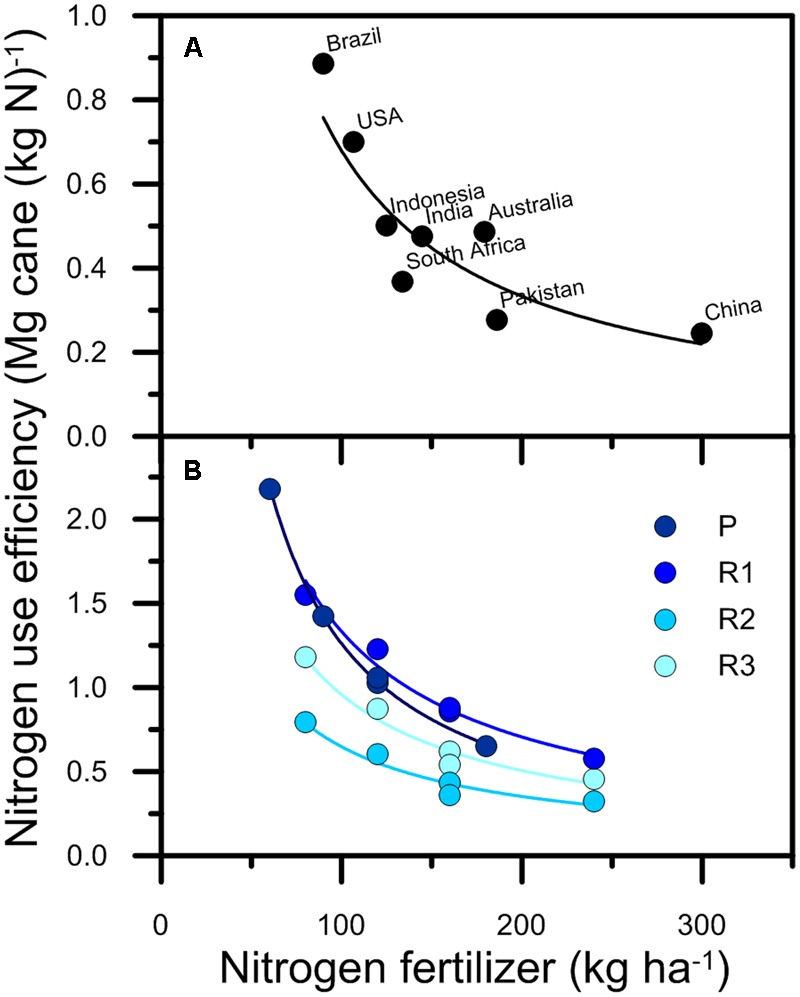
Nitrogen use efficiency of sugarcane production in **(A)** eight countries and **(B)** four crops [plant (P) and three ratoons (R1–R3)] in an Australian field experiment. In **(A)**, the results are averages derived from data on total production sugarcane production and nitrogen applied (Robinson et al., 2011). The results in **(B)** were derived from data given by Thorburn et al. (2003b).

Worldwide, NUE of sugarcane production ranges between approximately 0.25 and 0.9 Mg cane (kg N)^-1^ (**Figure 1A**) with countries that have higher average N fertilizer application rates having lower NUE. This sensitivity of NUE to N rate is well established (Ladha et al., 2005), as illustrated by N response experiments (**Figure 1B**). However, NUE also varies because crop yields vary in response to factors other than the amount of N fertilizer applied, so NUE values will be lower in years with lower yields. For example, in Australia average NUE over the last 20 years was 0.5 Mg cane (kg N)^-1^, but was 0.35 Mg cane (kg N)^-1^ in 2000 in response to low yields caused by widespread disease (Bell et al., 2014). At the scale of an individual field, NUE (at N application rates of 160 kg ha^-1^) varied from approximately 0.4 to 0.9 Mg cane (kg N)^-1^ across the three ratoons in the example shown in **Figure 1B**. The concerns over the environmental impact of N lost from Australian sugarcane production systems has prompted recent reviews of NUE (Wood et al., 2010; Bell et al., 2014). These reviews have produced general recommendations on pathways to better understand the determinants of NUE and opportunities for improving NUE such as increasing yield potential and exploring enhanced efficiency fertilizers (EEF). However, while the effect of N fertilizer application rate and crop size on NUE are known, the effect of other management factors such as timing of N fertilizer application, fallow management, or tillage is less clear.

The aim of this study was to establish for Australian sugarcane farming systems (1) the impacts of climate on factors determining NUE, (2) the range and drivers of NUE, and (3) regional variation in sugarcane N requirements. Such knowledge can prioritize ways to increase NUE within current production systems and/or refine management to increase NUE.

## Materials and Methods

### Overview

This study was conducted in four stages. Firstly, we simulated the response of sugarcane to N fertilizer at multiple sites to evaluate the model used in the study, adding to the previous testing of the model (Keating et al., 1999; Skocaj et al., 2013b; Meier and Thorburn, 2016). Secondly, we modeled simplified management systems to gain insights into the impacts of climate on factors determining cane yield, NUE, agronomic efficiency (AE), and N losses. Thirdly, we expanded this analysis to explore the range and drivers of NUE for multiple sugarcane management practices, climates, and soils that approach the scale of the entire sugarcane production area within Great Barrier Reef catchments. Furthermore, we analyzed the management practices within this simulation output that were associated with greatest NUE. Lastly, we determined the regional variation in sugarcane N requirement.

### Model Description

The Agricultural Production Systems sIMulator (APSIM; v.7.3 and v.7.7)^1^ is a deterministic, daily time-step modeling framework, capable of simulating plant, soil, climate and management interactions (Holzworth et al., 2014). APSIM was chosen for this study because of its established capability to simulate N dynamics in sugarcane production (as noted above) and N losses from sugarcane production systems through denitrification (Thorburn et al., 2010), leaching (Thorburn et al., 2011a), and runoff (Biggs et al., 2013). It also has the ability to represent a range of management practices relevant to this study, including: fertilizer split applications; crop rotations, specifically the effects of legumes on soil N in subsequent sugarcane crops (Park et al., 2010); and effects of tillage intensity and in-field traffic management on runoff and N losses (Thorburn et al., 2011a; Biggs et al., 2013).

For this study the APSIM model was configured to include modules for: soil N and carbon dynamics (APSIM—SoilN, Probert et al., 1998); soil and water dynamics (APSIM—SoilWat, Probert et al., 1998); surface organic matter (APSIM—SurfaceOM, Probert et al., 1998); and a range of crop modules (e.g., APSIM—Sugar, Keating et al., 1999). All modules are one dimensional and driven by meteorological data. Details of the modules are given in Supplementary Material 1.

### Simulating N Response for Model Evaluation

#### Sites

Five N response experiments were simulated that had been conducted on commercial sugarcane farms in north-eastern Australia with contrasting soils and climates (**Table 1**). Sites were located at Bundaberg, Mossman, Maryborough, Mulgrave, and Innisfail. The response of sugarcane yield to a range of N fertilizer rates had been measured at each site. Experiments ran for between 3 and 6 years, N fertilizer rates varied between 0 and 240 kg N ha^-1^, and there were between one and three replicates of each N fertilizer treatment. Further details about the experiments can be found in the references listed in **Table 1**.

**Table 1:** Details of soil texture, rainfall, experiment duration and reference to original experimental studies for the five N response experiments used for model evaluation.

Region	APSoil code	Soil texture (0.0–0.6 m)	Average rainfall (mm year^-1^)	Experiment duration	Reference
Bundaberg	bu-99	Sandy loam to sandy light clay	997	1996–2001	Thorburn et al., 2003b
Mossman	ms-01	Sandy clay	2,599	2003–2006	Thorburn et al., 2011b
Maryborough	mb-02	Sandy clay loam	995	2004–2007	Thorburn et al., 2011b
Mulgrave	ml-01	Sandy clay	2,082	2004–2008	Thorburn et al., 2011b
Innisfail	in-03	Light clay	3,623	2004–2008	Thorburn et al., 2011b

#### Parameterization of the APSIM Model

Values of parameters in APSIM came from three general sources (following Thorburn et al., 2011a): (1) derived from measurements at the sites; (2) standard values within the model, or some variation of those established in previous studies; or (3) calibration against measured values. Site measurements were used to determine values for the parameters drained upper limit, lower limit, saturation water contents, bulk density, soil organic carbon and N (Supplementary Tables S1–S5). The parameters controlling curve number, initial available water, rooting depth (Supplementary Table S6), the extent and severity of crop lodging, and water logging were determined by calibration against measured yield data. Initial values of soil mineral N were set to the values measured at the beginning of the experiment. Default values were used for all other crop and soil parameters, except for those that were modified based on previous sugarcane production system studies (Supplementary Table S7).

Simulation time frame depended on the experiment length (**Table 1**). Historical climate data was obtained from the Australian Bureau of Meteorology (via the SILO database^2^; Jeffrey et al., 2001) for meteorological stations close to each site (Supplementary Table S6).

A complete crop cycle (plant crop followed by multiple ratoons) was simulated for all sites. Farming operations including sowing and harvesting dates, and N fertilizer application rates and dates were specified to represent the actual operations that occurred on site (see references in **Table 1**).

### Impacts of Climate on Factors Determining NUE

Simulations were undertaken for two contrasting climates in which Australian sugarcane production occurs: (1) Tully, a high rainfall environment (average annual rainfall ∼4,000 mm), and (2) Mackay, a moderate rainfall environment (average annual rainfall ∼1,700 mm) where water stress is a more prominent limitation of sugarcane growth. At both locations, two soils of contrasting texture and soil carbon content were simulated (**Table 2**). In Tully, we used a gleyed Brown Dermosol (tu-02) and a coarser textured Yellow Dermosol (tu-03). The average carbon concentrations were 1.0 and 0.7%, respectively. In Mackay, we used a fine textured Vertosol (mk-02) and a coarser textured loam (mk-01). The average carbon concentrations (0.0–0.3 m) were 1.3 and 0.9%, respectively.

**Table 2:** Some details of the soils represented in the regional simulations.

Region	Soil code	Soil type	Reference
Bundaberg	bu-02	Red Dermosol	Verburg et al., 2001
	bu-11	Red Kandosol	Dawes et al., 2003
	bu-13	Redoxic Hydrosol	Dawes et al., 2003
Burdekin BRIA	bh-01	Medium clay	Thorburn et al., 2011a
	bh-02	Medium clay	Thorburn et al., 2011a
Burdekin Delta	bk-03	Silty clay loam/light clay	Thorburn et al., 2011a
	bk-04	Silty clay/coarse sand	Stewart et al., 2006
Mackay	mk-01	Loam	Macdonald et al., 2009;
			Denmead et al., 2010
	mk-02	Vertosol	Weier et al., 1998
	mk-03	Heavy clay loam	Masters et al., 2008
Tully	ba-01	Ferrosol	Meier et al., 2006
	ba-02	Hydrosol	Meier et al., 2006
	tu-02	Brown Dermosol	Cannon et al., 1992
	tu-03	Yellow Dermosol	Cannon et al., 1992

Yields and N losses were predicted for crops harvested each year from 1998 to 2004 following the approach taken by Thorburn et al. (2011c). This time period was selected as it included years with a large range in rainfall. In Mackay rainfall ranged from approximately 900 to >2,000 mm and at Tully from approximately 2,300 to 5,700 mm. The ability of the model to simulate yield responses was tested in Section “Simulating N Response for Model Evaluation” (and previous studies: Keating et al., 1999; Skocaj et al., 2013b; Meier and Thorburn, 2016). Simulation of denitrification and N leaching had been reported in previous studies (Thorburn et al., 2010, 2011a,c; Biggs et al., 2013).

A simplified production system was represented in the simulations to remove the confounding effects of factors such as harvesting time, crop class, and crop management (except for N fertilizer rate) on the predicted variables. All crops in the simulations were ratoon crops, harvested in mid-September, with crop residues retained on the soil surface and no tillage performed. N fertilizer was applied as urea, buried below the soil surface^3^, at a wide range of rates (up to 210 kg ha^-1^). The soil disturbance during application of fertilizer has little effect on infiltration or residue incorporation. The simulated crops at Mackay received no irrigation (as opposed to trying to reflect common practice in that region), to facilitate the comparison of rainfall variability between locations. Eighty years of sugarcane production was simulated prior to the first ratoon crop (i.e., harvested in 1998) to allow soil organic matter pools in the model to reach their dynamic equilibrium. To remove the confounding effects of the interactions between N fertilizer rate and soil organic matter build up or decline in the ratoon crop simulations, soil organic matter pool sizes, soil mineral N, soil water content, and surface residue mass were “reset” to the values that existed in the model at the start of the first ratoon crop in 1998.

The NUE was calculated for yields simulated at each N rate from:

(1) $$\text{NUE}=\frac{\text{Y}}{{\text{N}}_{\text{fert}}}$$

where Y is the crop yield and N_fert_ is the N fertilizer rate. In addition, AE was calculated from:

(2) $$\text{AE}=\frac{\left({\text{Y}}_{\text{N}}{\text{-Y}}_{\text{N0}}\right)}{{\text{N}}_{\text{fert}}}$$

where Y _N_ is the predicted yield at a particular N rate (kg ha^-1^) and Y _N0_ is the predicted yield with no N fertilizer applied.

Further analyses were performed to investigate the degree to which N losses to the environment might be limiting yields, especially in years of high rainfall. The relationships between rainfall, yields and N losses at a single N rate (180 kg ha^-1^) was investigated. This rate was chosen as it is one at which simulated yields were (or were almost) not N-limited.

### The Range and Drivers of NUE in Sugarcane Production

Sugarcane yields were simulated under a wide range of soils and climates in five contrasting regions, Bundaberg, Burdekin River Irrigation Area (BRIA), Burdekin Delta (DELTA), Mackay–Whitsunday, and Tully. Soils, climatic conditions, and management practices were simulated in factorial combinations within the five regions. The Burdekin region is commonly discussed as a single region, but was considered as two regions in this study because of the difference in soils and management, especially irrigation practices, between the BRIA and the DELTA (Thorburn et al., 2011a). Model parameters were collated from previous studies (**Table 2**) to represent important soil types in each region. There were two soil types in each of the BRIA and DELTA regions, three in the Bundaberg and Mackay regions and four in the Tully region (**Table 2**). Long-term historical climate data was obtained for representative meteorological stations in each region. For the Mackay region three stations were included (Eton, Plane Creek, and Proserpine).

A general sugarcane cropping cycle was defined for the simulations. Sugarcane was planted in autumn (April to June) and harvested 14–15 months later. Ratoon crops were harvested after approximately 13 months. There were three ratoons simulated for the Burdekin regions and four in other regions. The field was then fallowed for 6 months. In the Burdekin regions, if a legume grain crop was grown in the fallow (fallow management options are outlined below), sugarcane planting was delayed by 1 month and the plant crop was harvested after 13 months. All crop residues were retained on the surface after harvest except in the Burdekin where they were burnt, as is common practice in that region. All fertilizer N was applied as urea at a depth of 50 mm.

Crops were irrigated in the Bundaberg, Mackay, and Burdekin simulations. Irrigation was limited to a maximum of 375 mm crop^-1^ for Bundaberg and 100 mm crop^-1^ for Mackay–Whitsunday reflecting the limited water supply in these regions. The amount of water per irrigation was 37.5 mm for Bundaberg and 42.5 mm (equivalent to 50 mm with 85% irrigation efficiency for overhead irrigation) for Mackay–Whitsunday. In the two Burdekin regions, where irrigation supply is not limited, four different irrigation strategies were simulated. These gave a wide range in the amount of irrigation applied per crop (averaging 809, 1,537, 2,114, and 3,780 mm), achieved through spanning the typical differences in the amount of water applied in each irrigation (50, 80, 110, and 150 mm) and the frequency (approximately each 7–14 days) of irrigations following Thorburn et al. (2011a). Runoff from each irrigation was explicitly simulated based on soil hydrology parameters and antecedent soil conditions, rather than estimated from generic irrigation efficiency assumptions. The effects of water logging and lodging were included in the simulations, with the “rules” governing these processes derived from experience gained in simulating field experiments (Thorburn et al., 2011a; Skocaj et al., 2013b; Meier and Thorburn, 2016).

Management practices explored in the simulation were rates of N fertilizer, timing of N fertilizer application (relative to planting or ratooning), splitting N applications in plant crops, fallow management (bare, a ley legume or a grain legume), tillage (four levels, increasing in number and severity of operations) and in-field traffic management (controlled traffic or conventional). N fertilizer amounts applied came from either two recommendation “systems” or fixed amounts per crop. The two recommendation systems were “Six Easy Steps” (Schroeder et al., 2014) and N Replacement (Thorburn et al., 2011b). “Six Easy Steps” is the current recommended method for determining N fertilizer application rates, with the amount of recommended N varying according to district and soil type. N Replacement derives recommended N fertilizer rates from the actual yields previously grown. The fixed amounts simulated in Bundaberg, Mackay, and Tully were 40, 80, 160, 180, and 240 kg ha^-1^ crop^-1^ in ratoon crops, with 25% less N applied to plant crops. In the two Burdekin regions the fixed amounts were 40, 110, 180, and 320 kg ha^-1^ crop^-1^ with a similar reduction in plant crops.

To avoid having patterns in climate coincide with the patterns in the cropping cycle, simulations were started in each of 6 years, 1902–1907, and ran until 2011. Outputs were amalgamated over the six “start years.” Simulation outputs prior to 1927 were discarded to minimize the effect of non-equilibrium effects in the modeled system on simulation results. The combination of soils, climates, management practices, and start years resulted in 6.9 million sugarcane crops being simulated.

NUE was calculated for all crops. Further analysis was undertaken to identify which of the management factors included in the simulations (i.e., N fertilizer rate, timing of N fertilizer application, splitting N applications in plant crops, fallow management, tillage, and in-field traffic management) were associated with high NUE. To provide this information, the simulation results were statistically analyzed using “data mining” techniques (Supplementary Material 2) to associate the management practices with NUE.

### Regional Variation in Sugarcane N Requirement

The N requirement of sugarcane [kg N (Mg cane)^-1^, i.e., the inverse of NUE] is a fundamental parameter in systems for recommending N fertilizer application to sugarcane in Australia (Schroeder et al., 2014). It is multiplied by a yield goal to determine N fertilizer application rates. Currently a single N requirement value (1.4 kg N Mg^-1^ where yield potential is <100 Mg ha^-1^) is used across all sugarcane growing regions in Australia (Schroeder et al., 2014). This value was derived from simulations of the crop-to-crop variation in economic optimum rate of N for crops over a limited range of conditions, i.e., under a single climate (for the town of Ingham), single crop management system and a limited range of soils (Keating et al., 1997). The aim of this section of this study was to explore whether there was variability of the N requirement across regions under a wide range of conditions.

Determining the N fertilizer requirement consisted of two steps: (1) calculation of the economic optimum N rate from simulated N response curves, and (2) the derivation of the N requirement from the economic optimum N rates.

#### Economic Optimum N Rate

The economic optimum N rate was determined for N responses simulated for the climates, soils, and a sub-set of management factors described in Section “The Range and Drivers of NUE in Sugarcane Production,” following the general approach of Keating et al. (1997). A sub-set of the management factors was chosen to simplify the analyses because many of the management practices simulated (i.e., N fertilizer application date, splitting N applications in plant crops, tillage, and in-field traffic management) did not significantly affect simulated NUE (as described below). Thus, only a single level of these factors was included. As well, only bare fallows were simulated and only ratoon crops analyzed to avoid non-fertilizer N (e.g., from fallow legumes) affecting simulated N fertilizer requirement. The resultant management system approximated “common practice” (i.e., the “C-Class” system of van Grieken et al., 2010) with sugarcane growth simulated under a range of N fertilizer application rates. There were from ∼500 individual N response curves for each of the two Burdekin regions to ∼2,500 for Mackay.

A continuous yield-N response function is required to calculate the economic optimum N fertilizer rate (as that rate may have been different from those simulated). This continuous function was obtained by fitting empirical equations to the simulated yield-N response functions, as described in Supplementary Material 3.

The empirical equations were then used to calculate yield for N fertilizer rates from 25 to 300 kg ha^-1^ in 1 kg increments, then partial gross margins calculated (as described in Supplementary Material 4) at each N rate. The economic optimum N rate was defined as the N rate at which profitability was 99% of maximum. The cane yield at this optimal N rate was also calculated. The value of 99% of the maximum (rather than the maximum as used by Keating et al., 1997) was used to avoid numerical instability in the calculations.

#### Sugarcane N Requirement

The sugarcane N requirement [kg N (Mg cane^-1^)] is defined by the slope of the relationship between the economic optimum optimal N rate and the cane yield at that N rate. To determine the slope of this relationship, we fitted a linear quantile regression to the optimal N-cane yield results for each region. We fitted the regression to the 80th percentile: that is, for any given yield, there was an 80% chance that the optimal N rate was less than that implied by the quantile regression. This approach is somewhat different from that used by Keating et al. (1997). They used a qualitative approach to fit linear relationships that bounded all date (i.e., approximately equivalent to the “100th percentile” in our analysis).

## Results

### Simulating N Response for Model Evaluation

**FIGURE 2:**
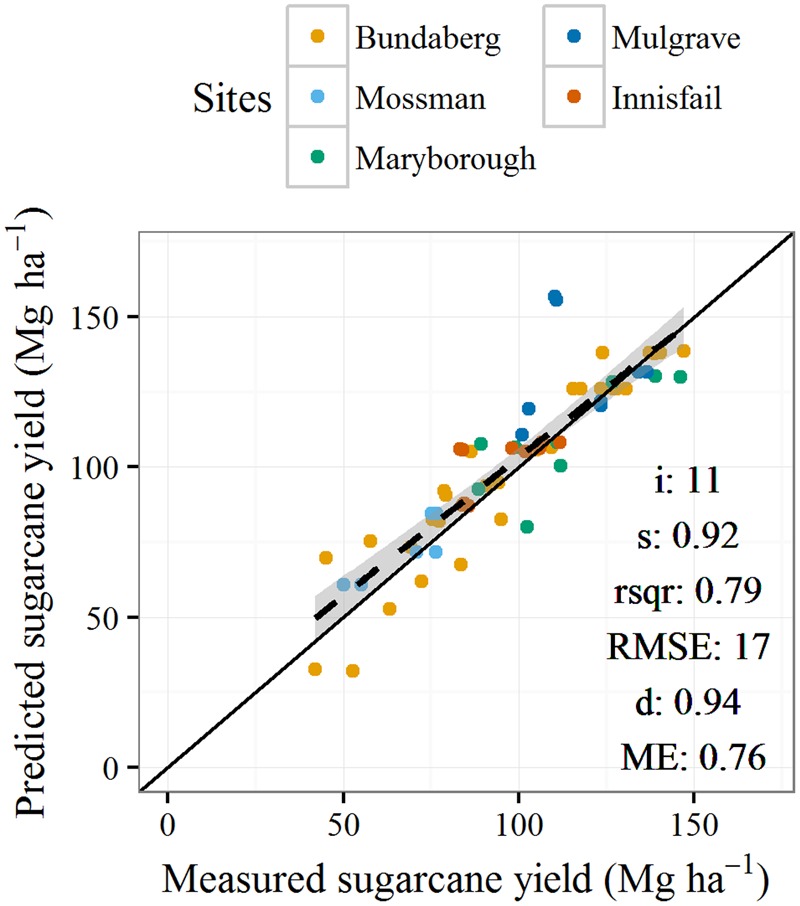
Measured (mean of replicates) and predicted sugarcane yield for five nitrogen response experiments (listed in **Table 1**). Nitrogen fertilizer application rates ranged between 0 and 240 kg N ha^-1^. Definitions of statistics presented in the figure are: i, intercept; s, slope; rsqr, *r*^2^; RSME, root mean square error; d, index of agreement (Willmott, 1982); ME, model efficiency (Nash and Sutcliffe, 1970).

**FIGURE 3:**
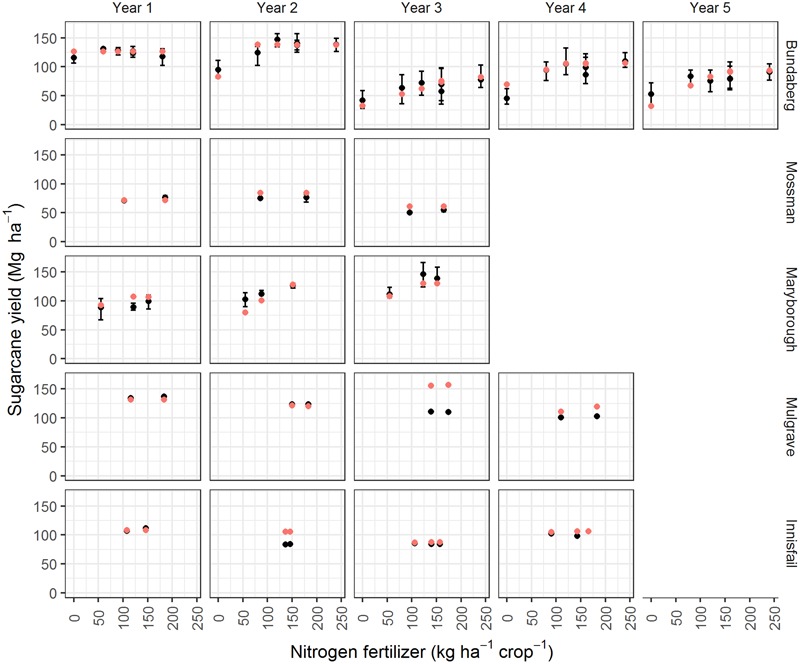
Measured (solid black points show the mean yield and “error bars” show the range of the replicates) and predicted (solid red points) sugarcane yields for five N response experiments (**Table 1**) conducted over 3–5 years. The experiments commenced in the following years: 1996 for Bundaberg; 2003 for Mossman; and 2004 for Maryborough, Mulgrave, and Innisfail.

Simulated sugarcane yield was well predicted across the sites (**Figure 2**) with statistics of prediction skill in line with similar studies (Keating et al., 1999; Thorburn et al., 2011a; Meier and Thorburn, 2016). The experiments simulated included situations where yields increased in response to increasing N applied (i.e., the Bundaberg and Maryborough sites, **Figure 3**) and where there was negligible N response. The simulations were able to capture these different responses generally within the error of measurement. In some instances measured yields were over predicted. The most notable of these was for the Mulgrave site in year 3 (**Figure 3**). There the crop was impacted by a cyclone (“Larry”) and severely lodged to an extent beyond that able to be captured in the model.

### Impacts of Climate on Factors Determining NUE

#### Annual Variations in Yields and N Parameters

**FIGURE 4:**
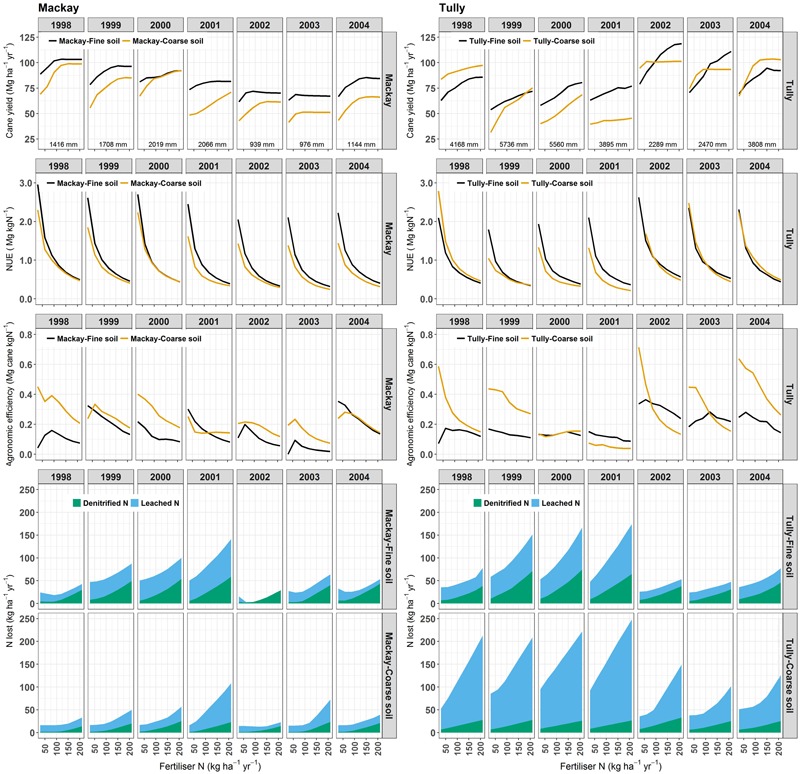
The response in sugarcane yields, nitrogen use efficiency (NUE), agronomic efficiency, and N lost through denitrification or leaching for ratoon crops (harvested at 12-month age) simulated under a wide range of nitrogen (N) fertilizer application rates with climate data from two locations for two soils of contrasting textures in each location. The total rainfall during each crop is listed under the yields, and details of the simulations are given in the text.

At both Tully and Mackay, yields were simulated to increase with increasing N fertilizer applied (**Figure 4**). The magnitude of the increase, however, was variable between locations, soils, and years. In many years, yields reached a “plateau” and did not increase with additional N. However, the N rate at which the plateau was reached was variable. In Tully, yields did not plateau in 1999 and 2000, the two wettest years, although the increase in yields with increasing N above 150 kg ha^-1^ was small in the fine textured soil in 2000.

**FIGURE 5:**
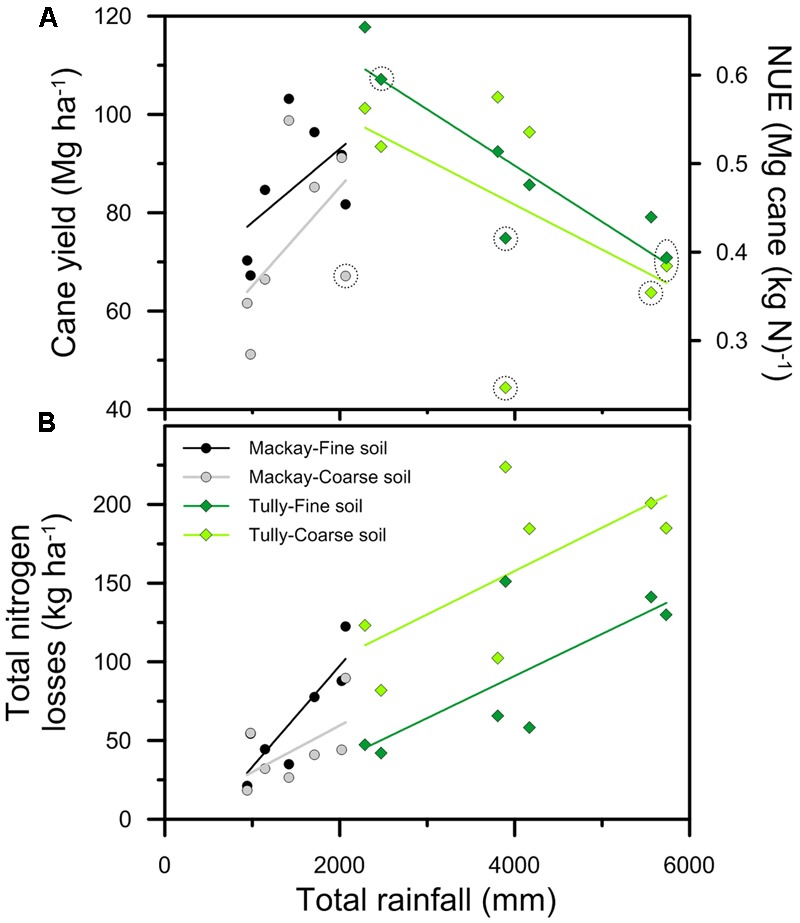
The relationship between total rainfall falling during each crop and **(A)** cane yield and nitrogen use efficiency (NUE), and **(B)** total nitrogen losses for crops simulated in **Figure 4** with 180 kg ha^-1^ of N fertilizer applied. The lines indicate the trends in yield and total nitrogen losses in response to rainfall at Mackay (gray and black) and Tully (light and dark green). The points circled indicate crops where yields simulated with 210 kg ha^-1^ of N fertilizer were more than 2% greater than yields at lower nitrogen rates. [NB: In **(A)**, NUE is derived from cane yield divided by 180 kg ha^-1^ of N.]

At high N rates (e.g., >150 kg ha^-1^) there was some impact of rainfall on simulated yields (**Figure 5A**). For Mackay, the lowest yields occurred in the two driest years (2002 and 2003). However, simulated yields could be high (e.g., >80 Mg ha^-1^) when the rainfall was ∼1,400 mm (1998) or ∼2,200 mm (2000).

The effect of soils on yield was more complex. In the Mackay simulations, yields with the fine textured soil were higher than with the coarse textured soil in 6 of the 7 years (**Figure 4**). The higher yields were generally due to the higher water holding capacity of the fine textured soil. The 2000 crop, when the yields were similar in both soil types (except at low N rates), not only received relatively high rainfall (the second highest rainfall in the simulations) but temporally well distributed rainfall such that soil water holding capacity was less important in determining the yield. The effect of soil texture was not as consistent in the Tully simulations. Yield simulated with the fine textured soil were higher than those with the course textured soil at all N rates in 2000 and 2001, at higher N rates in 2002 and 2003, but lower at most N rates in 1998 and 2004.

NUE ranged from between ∼ 0.5 and 3 Mg cane (kg N)^-1^ depending on fertilizer rate, soils, and climate. As expected, NUE decreased with increasing N fertilizer across all soils, locations, and years. AE generally declined when yields approached or reached a “plateau” and the numerator of Eq. 2 (*Y_N_* -*Y_N_*_0_) changed little with increasing N rate. Maximum AE values varied between years and locations, from close to 0.4 Mg cane (kg N)^-1^ at Mackay in 1998, 2000, and 2004, to >0.6 Mg cane (kg N)^-1^ at Tully in 1998, 2002, and 2004 (**Figure 4**). These high values occurred at low N rates, as expected, and generally in the coarse soils (Mackay in 2004 being the exception). Further, maximum AE values were higher in the coarse textured soils in 5 of the 7 years at both sites. The coarse textured soils had lower soil organic matter content than the fine textured soil (as described above), and so the contribution of mineralized N to crop N requirements was lower in these soils, explaining the greater relative response to N fertilizer. At high N rates, AE values in the different textured soils were more similar (and also lower than at low N rates), as expected because the supply of N from fertilizer over shadowed different amounts of N from mineralized soil carbon in the soils investigated. In fact, in Tully in 2002 and 2003, AE values at high N rates were higher in the fine textured soil as yield were still responding to additional N applications in this soil, but not the coarse textured soil. In Tully, 2002 and 2003 were the two driest years of those simulated and the lower soil water holding capacity of the coarse textured soil increased water stress in the simulated crops (data not shown). Thus water stress was the primary limit to yields and adding high amounts of N fertilizer in these years was agronomically “inefficient.” These results suggest that AE is a complex parameter and it is difficult to attribute a particular AE value or difference in AE values between different situations to a single causal factor.

Like yields, simulated N losses generally increased with increasing N fertilizer applied (**Figure 4**). Losses were also variable between locations, soils, and years. Losses were generally higher in the fine than coarse textured soil for Mackay, but higher in the coarse textured soil for Tully. Losses were also generally related to rainfall, for example, being highest (at the highest N rates) in 2001. However, while 2001 was a “wet” year, it was not the year with highest rainfall at either Mackay or Tully. A greater proportion of N was lost generally by denitrification than leaching from the fine textured soils at both locations, although in all cases losses by denitrification were lower than leaching at low N rates. Thus denitrification was more sensitive to N rate than leaching. However, leaching was more responsive to climate than denitrification such that losses by leaching relative to denitrification were higher in years with greater losses (e.g., 2001) the in years with lower losses (e.g., 2002).

#### Potential for Yields to be N-Limited in High Rainfall Years

As stated above, simulated yields tended to increase with rainfall at Mackay, but decrease at Tully (**Figure 5A**). As yields changed, NUE also changed (given that 180 kg ha^-1^ of N fertilizer was applied in all simulations). Thus NUE tended to increase with increasing rainfall at Mackay, but decrease at Tully, indicating the strong climatic impact on NUE.

While yields (and NUE) were affected by rainfall, the relationship between rainfall and yields is complex, as noted above. This complexity results in the relationship between yield and total rainfall only being significant (*P* < 0.05) in the simulations of the fine textured soil with the Tully climate. In contrast, N losses increased with increasing rainfall at both locations (**Figure 5B**). The relationships were significant for both soils in the Tully simulations (*P* < 0.10) and the fine textured soil with the Mackay climate (*P* < 0.05).

Both yields (**Figure 5A**) and N losses (**Figure 5B**) tended to increase with increasing rainfall in the Mackay simulations suggesting that rainfall limited cane yields more than N. If so, applying larger amounts of N fertilizer would not have notably increased yields. This was the case in 13 of the 14 crops simulated: only in the coarse soil in the wettest year simulated (2001, **Figure 4**) was applying 30 kg ha^-1^ more N fertilizer (i.e., the 210 kg ha^-1^ N rate, c.f. 180 kg ha^-1^) predicted to notably (i.e., by more than 2%) increase yields; i.e., this was the only crop simulated where yields were N-limited at 180 kg N ha^-1^.

**FIGURE 6:**
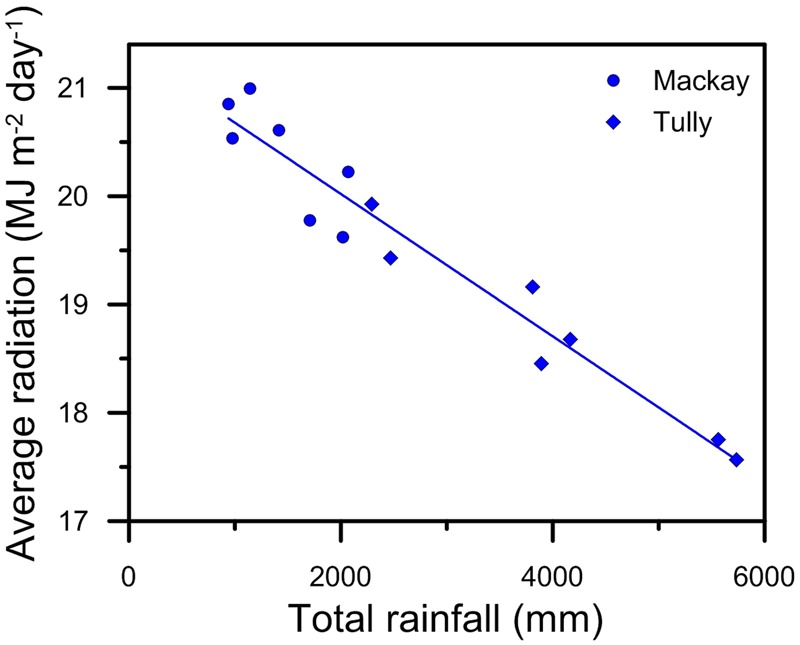
The variation in average daily radiation with rainfall for the crops simulated in **Figure 5**.

The relationship between rainfall and yield was different in the Tully simulations, a region where the high rainfall means crop water stress is less of a limitation to growth than in Mackay. In the Tully simulations, the higher rainfall resulted in lower yields (**Figure 5A**) and higher N losses (**Figure 5B**). It is tempting to assign “cause and effect” to this correlation. However, there are climate factors, importantly radiation (**Figure 6**), that are related to rainfall and affect sugarcane growth independent of N dynamics so it is unclear whether the decline in yields with increasing rainfall (**Figure 5A**) were due to increased N losses or radiation limits to growth. The limitation of N on crop growth in the simulations is indicated by the increase in yield from the application of larger amounts of N fertilizer. Of the 14 crops simulated for Tully, six had yields increase by more than 2% with the application of 30 kg ha^-1^ more N fertilizer (**Figure 5A**) indicating that these crops were N-limited at 180 kg N ha^-1^. Of these six N-limited crops, three occurred in the two highest rainfall years (1999 and 2000), although another crop in these years (the fine textured soil in 2000) was not N-limited. Of the other three N-limited crops, two occurred in a year (2001) with close to average rainfall and one in a relative dry year (2003). Thus, while crops simulated at Tully tended to be N-limited in the wettest years, that was not an inevitable situation; N limitations could occur in any year. Rainfall distribution is an important factor as well as amount (Skocaj and Everingham, 2014; Everingham et al., 2016).

### The Range and Drivers of NUE in Sugarcane Production

#### Range in Simulated NUE

**FIGURE 7:**
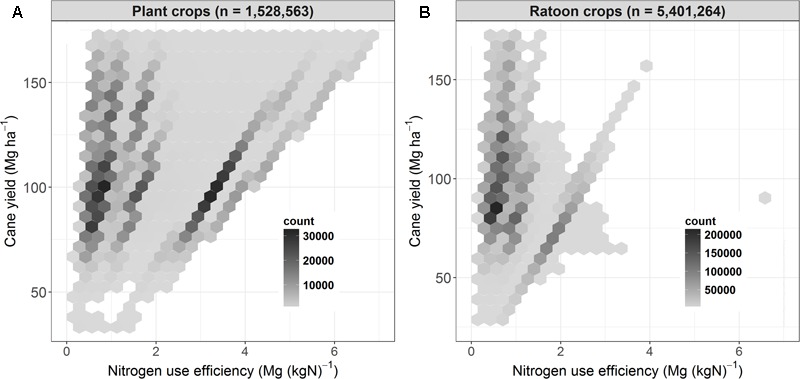
Yield as a function of nitrogen use efficiency (yield produced/fertilizer N applied) for sugarcane plant **(A)** and ratoon **(B)** crops simulated across regions, soil types, and management practices over 84 years (1928–2011). Hexagons contain all the data points located in that region of the figure. The number of points plotted within each hexagon is indicated by the shade of gray of the hexagon (darker shades represent a higher number of points).

There was a wide range of NUE within the simulated sugarcane crops, with values of ∼0.3 Mg cane (kg N)^-1^ in some simulations where yields were small (i.e., <50 Mg ha^-1^), to >4 (ratoon crops) or 5 (plant crops) Mg cane (kg N)^-1^ where yields were high and N fertilizer inputs low (**Figure 7**). This high variation results from the numerous interactions between climate (as illustrated above, **Figure 5A**), soils and management to produce a wide range of yields that, in many cases, were independent of the amount of N applied. For example, the linear patterns apparent in **Figure 7** are the result of different yields in different years (coming from climate, soils, and management interactions) when a constant N rate (e.g., 140 kg ha^-1^) was applied to crops in the simulations.

High NUE values [i.e., >2 Mg cane (kg N)^-1^] dominantly occurred in plant crops (**Figure 7A**), representing the low N fertilizer inputs to plant crops that occurs with some of the N management systems simulated (e.g., reducing N fertilizer applied following a legume fallow). However, most plant crops had NUE values of 0.7–1.8 Mg cane (kg N)^-1^ occurring at yields of 70–150 Mg ha^-1^. In comparison, the most common NUE values in ratoon crops were 0.4–1.2 Mg cane (kg N)^-1^ occurring at yields of 70–110 Mg cane ha^-1^ (**Figure 7B**).

There was a trend for NUE to increase with increasing yield in plant crops. In ratoon crops, however, the highest yields (e.g., >120 Mg ha^-1^) were generally associated with a NUE value of 0.8–1.2 Mg cane (kg N)^-1^, whereas the highest NUE values [i.e., >2 Mg cane (kg N)^-1^] were mainly associated with yields <100 Mg ha^-1^.

#### Management Factors That Influence NUE

**FIGURE 8:**
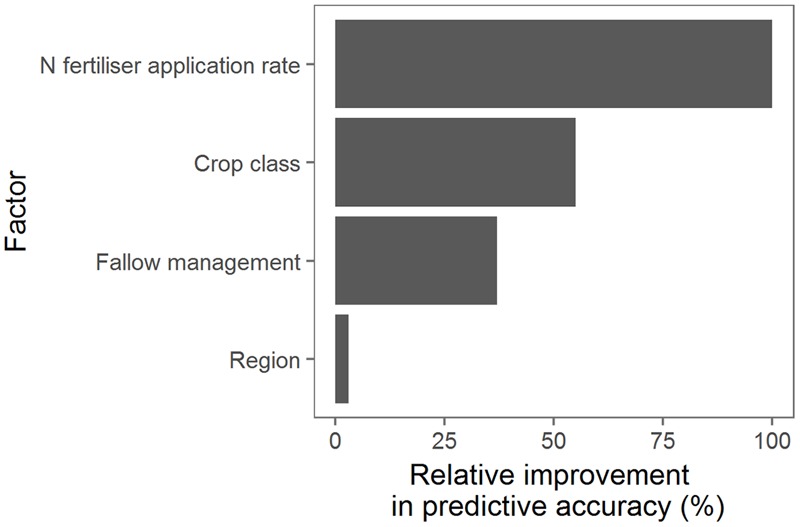
Ranking of factors (primary and surrogate) determining nitrogen use efficiency [Mg cane (kg N)^-1^] predicted for 6.9 million sugarcane crops simulated under a wide range of management practices, soils and climates over 84 years. The ranking of the factors was based on their improvement in predictive accuracy across the whole regression tree (**Figure 9**) and were ranked relative to the most influential factor. Only the four factors shown in this figure improved the predictive accuracy in the pruned regression tree.

**FIGURE 9:**
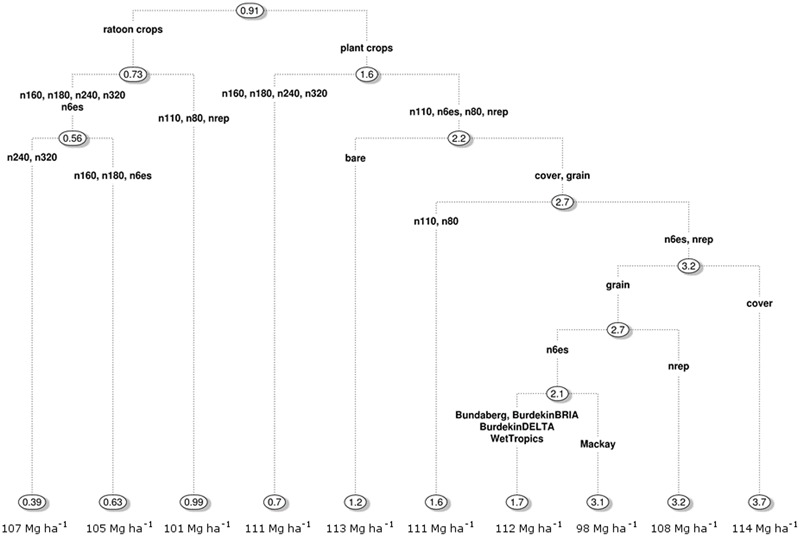
A pruned regression tree analysis of the factors determining nitrogen use efficiency [Mg cane (kg N)^-1^] predicted for 6.9 million sugarcane crops simulated under a wide range of management practices, soils and climates over 84 years. Labels are explained in Section “Management Factors That Influence NUE.” Average yields for the branches of the regression tree are displayed across the bottom of the figure.

Four variables explained 79% of the variation in NUE across simulated sugarcane crops; N application rate, crop class, fallow management, and the region in which the simulated crops were located. The N rate provides the greatest improvement in prediction accuracy, followed in order by the other three variables (**Figure 8**). The other variables in the analysis (timing of N fertilizer application, splitting N applications in plant crops, fallow management, tillage, in-field traffic management, climate, and soil type) improved prediction by 1% compared with N rate (data not shown), and so were not included in the regression tree (**Figure 9**) to avoid over fitting. While N rate gave the greatest improvement in prediction accuracy, it was not the variable that dictated the first split in the regression tree (for reasons explained in Supplementary Material 2). That variable was crop class.

Across simulated sugarcane crops, the average NUE was 0.91 Mg cane (kg N)^-1^ (**Figure 9**). The NUE was lower in ratoon crops [averaging 0.73 Mg cane (kg N)^-1^] than plant crops [averaging 1.6 Mg cane (kg N)^-1^]. For both ratoon and plant crops, the most influential factor on NUE was N fertilizer application rate. For ratoon crops, three main groupings of N fertilizer application rates emerged from the analysis: The first grouping came from applying fixed N rates of 240 or 320 kg N ha^-1^ crop^-1^ (denoted n240 and n320 in **Figure 9**) to every ratoon crop and resulted in the lowest average ratoon crop NUE [0.39 Mg cane (kg N)^-1^]. The second grouping came from applying between 160 or 180 kg N ha^-1^ crop^-1^ (n160 and n180) or using the “Six Easy Steps” system (n6es) which increased average NUE to 0.63 Mg cane (kg N)^-1^. The third grouping of N rates for ratoon crops came from applying <110 kg N crop^-1^ (n110 and n80) or using the N Replacement system (nrep) and resulted in an average NUE 0.99 Mg cane (kg N)^-1^. However, as N rates decreased and NUE increased, average cane yields decreased; from 107 Mg ha^-1^ for the highest grouping of N management to 105 and 101 Mg ha^-1^ for the second and third lowest groupings. For ratoon crops, none of the other management factors (timing of N fertilizer application, fallow management, tillage, or in-field traffic management) significantly influenced NUE.

For plant crops, there were only two groupings for N fertilizer rates: (1) applying between 160 and 320 kg N ha^-1^ crop^-1^, and (2) applying <110 kg N crop^-1^, using the “Six Easy Steps” or N Replacement systems (**Figure 9**). The higher N rate grouping had an average NUE of 0.7 Mg cane (kg N)^-1^ and yield of 111 Mg ha^-1^. For the lower N rate grouping, NUE was most influenced by management of the preceding fallow, with bare fallow associated with lower NUE [average of 1.2 Mg cane (kg N)^-1^] than fallows with a legume grown either as a grain or a cover crop. For the plant crops preceded with a legume fallow, the average NUE depended on multiple interactions between the specific N rates, type of legume (“grain” or “cover crop”), and region. For the different groupings, average NUE ranged from 1.6 to 3.7 Mg cane (kg N)^-1^. Unlike the situation with ratoon crops, there was no trend in average cane yield between the different groupings, with the highest average cane yield (114 Mg ha^-1^) occurring in the grouping with the highest average NUE. The lack of correlation between cane yield and NUE was affected by the N contained in the legume crops, which was available to the plant crop but not included in the calculation of NUE. As with the ratoon crops, other management factors (splitting N fertilizer applications, tillage or in-field traffic management) did not significantly influence NUE.

The data mining analysis was also conducted on simulations for each individual region (data not shown). As in the combined regions analysis, the factors most affecting average NUE were crop class (plant vs. ratoon crops), N rate and fallow management. However, there were differences in order of importance of these factors. For example, N rate was the primary determinant of NUE in Tully and Bundaberg, whereas it was crop class in the other regions. In the DELTA region, soil type was also a significant factor. The average NUE (across all crops) also differed between regions, being highest in the two Burdekin regions (which had the highest average yields) and lowest in Bundaberg (the lowest average yields). As with the analysis of the combined regions, timing of N fertilizer application, splitting N in plant crops, tillage, and in-field traffic management did not significantly influence NUE.

#### Comparison of Results with Industry Average Values

An approach to examine the accuracy of our simulations is to compare our simulated NUE values with those that occur in Australian sugarcane production systems. Applying between 160 or 180 kg N ha^-1^ crop^-1^ or using the “Six Easy Steps” system encompasses typical N fertilizer management practices in Australian sugarcane production (Schroeder et al., 2014). The average NUE predicted for these practices [0.63 Mg cane (kg N)^-1^] agrees well with an industry-wide average value (2004–2014) of 0.54 Mg cane (kg N)^-1^ (Bell et al., 2014) considering the assumptions in the simulations. Higher simulated NUE values are expected given the absence of many yield-reducing factors (e.g., weeds, pests, and diseases) in the simulations result in higher yields and hence lower NUE. For example, if simulated yields were reduced by 15% the predicted NUE would be the same as the industry-wide average value.

### Regional Variation in Sugarcane N Requirement

**FIGURE 10:**
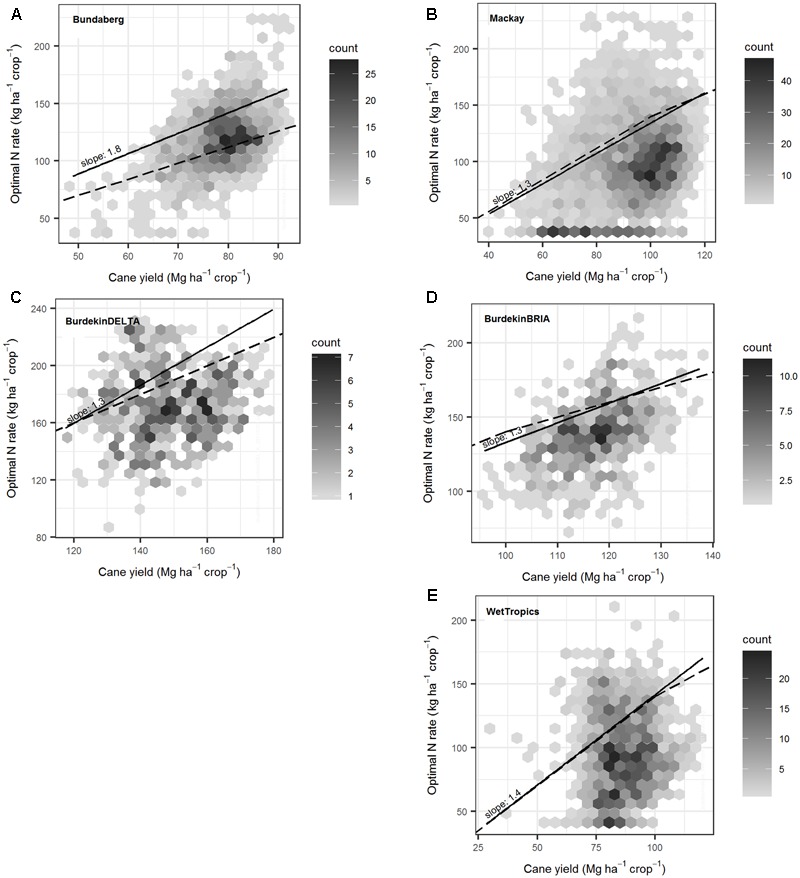
The variation in optimal nitrogen (N; i.e., the N fertilizer rates at which profitability is 99% of maximum) with yield predicted for ratoon crops over 84 years (1928–2011) in five regions **(A–E)**. In all simulations, crops were managed under common practices. In each plot, hexagons contain all the data points located in that region of the figure. The number of points plotted within each hexagon is indicated by the shade of gray of the hexagon (darker shades represent a higher number of points). The solid line indicates the quantile regression fitted to the 80th percentile (with the slope shown), and the dashed line is the general N application rate guideline for sugarcane (Keating et al., 1997).

For any given sugarcane yield our analysis suggests there can be a wide range in economic optimum N rates (**Figure 10**). In the crops of the Wet Tropics, for example, the economic optimum N rates ranged from <50 to >150 kg ha^-1^ for yields of 60–100 Mg ha^-1^. The variations in economic optimum N rates was caused by variations in soils, management and year-to-year climatic variability.

There was regional variation in the N fertilizer requirement (defined by the slope of the quantile regression between the economic optimum optimal N rate and the cane yield, **Figure 10**). The N fertilizer requirement was 1.3 kg N (Mg cane)^-1^ in the Mackay and two Burdekin regions, 1.4 kg N (Mg cane)^-1^ in the Wet Tropics and 1.8 kg N (Mg cane)^-1^ in Bundaberg (**Figure 10**).

## Discussion

Improved understanding of the factors that influence NUE and approaches to increase NUE are important in cropping systems. This is particularly true for sugarcane production in north-eastern Australia where N losses from sugarcane directly affect the health of Great Barrier Reef ecosystems (Brodie et al., 2012, 2013; Thorburn et al., 2013b; Kroon et al., 2016). This study provides the first comprehensive assessment of NUE in sugarcane production in this region, and the responsiveness of NUE to different soils, climate, and the management practices common in the region. We showed that the interactions between climate, soils, and management produce a wide range of simulated NUE in Australian sugarcane production systems (**Figure 7**), ranging from ∼0.3 Mg cane (kg N)^-1^ where yields were low (i.e., <50 Mg ha^-1^) to >5 Mg cane (kg N)^-1^ in plant crops where yields were high and N fertilizer inputs low. This range suggests there is scope to increase NUE from the recent average values of ∼0.5 Mg cane (kg N)^-1^ (Bell et al., 2014). However, of the wide range of management practices simulated, the only practice significantly influencing NUE in ratoon crops, the dominant class of crops in sugarcane crop cycles, was N fertilizer application rate (**Figure 9**). N fertilizer rate was also an important factor determining NUE in plant crops, although for plant crops receiving low amounts of N fertilizer (i.e., <110 kg ha^-1^ crop^-1^) NUE was also affected by the management of the preceding fallow. Thus it is clear that priority should be placed on optimizing N application rate, then fallow management for improving NUE in Australian sugarcane production systems.

Substantial funding has been given to Australian sugarcane farmers through government grants to adopt management practices to reduce N losses from their farms (Kroon et al., 2016). Much of this funding has gone into facilitating subsurface application of fertilizer, implementation of controlled traffic farming and/or planting legumes in fallows (Thorburn et al., 2011d). Of these, only planting legumes was predicted to significantly increase NUE compared to common practice (bare fallows, **Figure 9**) and, as noted above, fallow management was only significant in plant crops receiving low amounts of N fertilizer (i.e., <110 kg ha^-1^ crop^-1^). While the incorporation of legume crops in fallows is a positive result, it still does not diminish the importance of N fertilizer management in increasing NUE, for two reasons. Firstly, even if legumes were grown during fallows, N fertilizer management was still predicted to be important, with NUE more than doubling (1.6–3.7, **Figure 9**) with the best practice N fertilizer management simulated. Secondly, fallow management only significantly affects plant crops which account for only ∼20% of sugarcane crops, thus reducing the impact of this management practice on total N losses from sugarcane production. This small (in area) and variable effect of improved fallow management may have contributed to the generally small reduction in N discharged to the Great Barrier Reef Lagoon despite substantial government action (Kroon et al., 2016). Thus it will be important to focus on N fertilizer rates to reduce N loses from Australian sugarcane production systems.

Given the importance of N fertilizer rates in determining NUE, it is valuable to consider the N fertilizer recommendation systems used in Australian sugarcane production and consider scope for their improvement. The N recommendation systems have evolved from simple recommendations of a single rate for a wide range of soils and regions to systems based on partial N balances for crops (Schroeder et al., 2014). In the current system supported by the sugarcane industry, known as “Six Easy Steps” (Schroeder et al., 2014), the recommended rate of N fertilizer (kg ha^-1^) is the product of the target yield (Mg ha^-1^) and the N requirement of sugarcane (kg N Mg^-1^, i.e., the inverse of NUE), less the estimated N supply to crops from organic sources (e.g., mineralization of organic N from soil organic matter or crop residues). Improved recommendations from this system would thus result from improved knowledge of the target yield, N requirement and/or N supplied from organic sources. Of these factors, the yield target has received recent attention. The system was developed and tested based on the yield target being a regional yield potential (Schroeder et al., 2014) and there has been discussion about whether the target could be set at a smaller scale (e.g., farm or field; Bell, 2014) given that the district level target is rarely reached (Schroeder et al., 2010). Regardless of the scale being considered, a problem with selecting a yield target is that sugarcane yields in Australia are highly variable in both space (i.e., within and between regions) and time (between years), as illustrated by the results in **Figure 4**. One reason for the variability is the substantial seasonal climate variability experienced in sugarcane producing regions (Everingham et al., 2007). Another cause of the variability is the substantial range in harvested crop age (e.g., 8–24 months) and hence crop size. Developing ways to account for yield variation may better match N applications to crop productive potential and hence increase NUE.

The N requirement of sugarcane has received much less attention. Currently a single value, 1.4 kg N Mg^-1^ (where yield potential is <100 Mg ha^-1^), is used by the industry for all sugarcane production (Schroeder et al., 2014). This value was based on work by Keating et al. (1997), who used a small number of simulated N response curves under a single climate and a limited range of management practices. We have shown that there is regional variation in N fertilizer requirement (**Figure 10**) that could possibly make N fertilizer recommendations more specific. Our analysis combined different soil types in each region and thus more detailed analyses may also reveal soil- or management practice-specific N fertilizer requirements. Such results would be the potential path toward reducing the generality of recommendations. The quantitative approaches used in this study to derive N fertilizer requirement from simulated N response curves also provides a more robust basis on which to justify sugarcane N recommendation systems in the face of the environmental concerns confronting the Australian sugarcane industry (Brodie et al., 2013; Kroon et al., 2016).

While we have identified the factors that currently have the greatest influence on NUE in Australian sugarcane production systems, it is also valuable to consider options for increasing NUE in the future. Given the substantial impact that seasonal climate has on both yield and yield response to N fertilizer in Australia (**Figure 4**), seasonal climate forecasting may help address climate variability and improve N recommendations (Thorburn et al., 2011c; Skocaj et al., 2013a; Skocaj, 2015). In the Tully area, the size of the sugarcane crop (relative to median yields) can be predicted early in the growing season (i.e., ∼9 months prior to harvest) from a combination of observed climate, forecast climate and APSIM modeling (Everingham et al., 2016). These variables also influence yield response to N fertilizer (Thorburn et al., 2011c; Skocaj, 2015) indicating the potential for seasonal climate forecasting to improve N fertilizer management. Our results show that there are likely to be region-specific, soil × climate interactions for yields and yield responses to N (**Figure 4**). So any advances in using seasonal climate forecasting to improve N management will need to account for soil, crop, and climate interactions. Such an approach could also account for region-specific (e.g., **Figure 10**), or even soil-specific variations in N fertilizer requirement. A decision support system (DSS) may be an ideal platform for delivering soil specific predictions of optimum N fertilizer rates based on seasonal climate forecasts.

DSS have been developed to improve N fertilizer management in a range of crops (Ladha et al., 2005). An example is YieldProphet^®^ (Hochman et al., 2009)^4^, a system based on the APSIM model and used widely in the Australia grains industry to guide tactical, in-season N fertilizer management. Mechanistic models of N cycling in sugarcane cropping systems have not yet been harnessed to guide N fertilizer management decisions for sugar, although the concept of a DSS for improving N management both before and within a growing season has received some attention in Australia (Thorburn et al., 2011c). Application of a DSS for sugarcane may allow the provision to farmers of N fertilizer recommendations that respond to the soil, climatic and management factors that drive variability in N responses, and so facilitate site specific N fertilizer management and increased NUE.

EEF potentially have a role to play in increasing NUE in cropping systems (Chen et al., 2008). EEF are being trialed in the Australia sugarcane cropping systems; however, results have been inconclusive (Verburg et al., 2016). The variation in results are not surprising given experiences in other cropping systems. Hatfield and Venterea (2014) attributed the mixed efficacy of EEF in USA cropping systems to climatic variation during the growing season. If this conclusion is widely applicable, it suggests that the high degree of climate variability experienced in Australian sugarcane cropping systems (Everingham et al., 2007) is likely to result in inconsistent results. DSS that can incorporate climate variability may have a role to play in increasing the efficacy of EEF in these cropping systems.

Our simulation results show that complex interrelationships exist between climates, crop growth, N fertilizer rates, and N losses to the environment, even with a highly simplified representation of sugarcane crop management. The model used in this study, APSIM, is well tested on Australian sugarcane production systems (**Figures 2, 3**; Keating et al., 1999; Thorburn et al., 2010, 2011a,c, 2013a; Biggs et al., 2013; Skocaj et al., 2013b; Meier and Thorburn, 2016) and elsewhere (Marin et al., 2014, 2015; De Oliveira et al., 2016). However, it will be valuable to empirically test the major conclusions from this study.

## Author Contributions

PT conceived and designed the study, analyzed the data, and wrote the paper. JB and JP performed the simulations, analyzed the data, and wrote the paper. EM performed the simulations and analyzed the data. KV and DS wrote the paper.

## Conflict of Interest Statement

The authors declare that the research was conducted in the absence of any commercial or financial relationships that could be construed as a potential conflict of interest.

## Footnotes

1 http://www.apsim.info/

2 https://www.longpaddock.qld.gov.au/silo/

3 Fertilizer is applied below the soil surface with a “stool splitter,” an implement that has a coulter to cut into the soil at the base of the cane plant allowing fertilizer to be dropped into the base of the cut.

4 www.yieldprophet.com.au
