# Supplementary material for: Prioritizing Crop Management to Increase Nitrogen Use Efficiency in Australian Sugarcane Crops
Source: Front Plant Sci. 2017 Sep 5;8:1504. doi: 10.3389/fpls.2017.01504 (PMC5591824; doi:10.3389/fpls.2017.01504)
Supplement: Supplementary file 1 [file Data_Sheet_1.pdf]

Supplementary Material

Prioritizing crop management to increase nitrogen use efficiency in Australian sugarcane crops

Peter J. Thorburn*, Jody S. Biggs, Jeda Palmer, Elizabeth A. Meier, Kirsten Verburg, Danielle M. Skocaj

*** Correspondence:** Peter J. Thorburn: peter.thorburn@csiro.au

1 Details of the modules used in the APSIM model for this study

The APSIM-SoilN module represents the organic matter in soil layers as several discrete pools, each of which is characterized by a rate of decomposition that is sensitive to soil temperature and soil moisture (Probert et al., 1998). Fresh organic matter (FOM) is derived from senescing roots or the incorporation of surface residues. Soil organic matter comprises two pools, the BIOM pool representing microbial biomass with a high rate of decomposition and the HUM pool, representing the humus fraction of soil organic carbon (SOC). A portion of the HUM pool is considered to be inert (Probert et al., 1998). SOC content of each layer and its distribution between the BIOM and HUM pools are required as inputs to initialize the model. Following initialization, the value of these variables is calculated daily in response to simulated inputs and outputs (via decomposition) of each pool in each soil layer. The soil C:N ratio, also an initial input, determines the corresponding N in each of the pools. Total soil C:N can vary during the simulations in response to changes in the relative size of the carbon pools. Decomposition results in emission of carbon dioxide, synthesis of carbon into the different pools and mineralization or immobilization of soil mineral N (SMN). Decomposition of FOM with a high C:N ratio will create a demand for N, causing immobilization (Probert et al., 1998). SMN in excess of that needed to form new soil organic matter is mineralized as ammonium-N. Ammonium-N is converted to nitrate-N, which in turn can be lost via leaching, runoff or denitrification. The last process results in the production of nitrous oxide, as does nitrification.

In this study the APSIM-SoilWat module used a tipping bucket approach to simulate water movement between soil layers (Probert et al., 1998). Water is simulated to enter the soil from rainfall and/or irrigation and is partitioned to runoff and infiltration by the curve number approach (Holzworth et al., 2014; Probert et al., 1998). Cultivation and plant residue on the soil surface impacts curve number and thus affects runoff and infiltration, and also affects evaporation. Critical parameters that define soil water status in each soil layer are air-dry, drained upper limit, lower limit, and saturation. The water holding capacity of the soil, which is the total amount of water in the soil that a plant can access, is defined as the soil water held between the drained upper limit and the lower limit.

In common with all APSIM crop modules, the APSIM-Sugar module uses intercepted radiation to accumulate biomass, which is partitioned to various plant components such as roots, stalk or leaf (Keating et al., 1999). Biomass accumulation is responsive to radiation and temperature, as well as to

water and N deficiencies (Keating et al., 1999). Sugarcane can exhibit luxury uptake of N (Muchow and Robertson, 1994). This is represented in APSIM-Sugar by the difference between maximum and critical N concentrations (Keating et al., 1999).

Farming operations such as sowing, harvesting, fertilization, irrigation, and tillage are specified in APSIM-MANAGER or APSIM-OPERATIONS modules.

More details of the processes and modules described in this section can be found at http://www.apsim.info/.

Table S1. APSIM soil and crop parameters used for the Bundaberg site.

Depth (m)	0.0-0.10	0.10-0.20	0.20-0.40	0.40-0.60	0.60-0.90	0.90-1.20	1.20-1.50	1.50-1.75	1.75-2.00
**Organic C (%)**	0.82	0.58	0.34	0.23	0.15	0.12	0.11	0.11	0.11
**pH (water)**	6.25	6.25	6.25	6.9	7.3	7.5	7.75	7.75	7.75
**FBIOM**	0.035	0.028	0.018	0.015	0.015	0.01	0.01	0.01	0.01
**FINERT**	0.06	0.1	0.2	0.5	0.75	0.9	0.99	0.99	0.99
**Bulk density ($g\ cm^{-3}$)**	1.33	1.47	1.63	1.57	1.55	1.63	1.63	1.59	1.59
**LL15 & sugar LL ($mm\ mm^{-1}$)**	0.10	0.10	0.11	0.12	0.14	0.15	0.16	0.16	0.16
**DUL ($mm\ mm^{-1}$)**	0.17	0.17	0.17	0.18	0.20	0.23	0.24	0.24	0.24
**SAT ($mm\ mm^{-1}$)**	0.33	0.31	0.29	0.28	0.29	0.31	0.33	0.33	0.33
**Sugar KL**	0.1	0.1	0.1	0.1	0.08	0.08	0.04	0.04	0.04
**Sugar XF**	0.2	0.2	0.2	0.2	0.2	0.2	0.2	0.5	0
**SWCON**	0.4	0.4	0.4	0.4	0.3	0.3	0.3	0.3	0.3
**NO_3-N ($kg\ ha^{-1}$)**	7.5	7.5	3.3	2.1	3.2	3.9	6.4	5.3	5.3
**NH_4-N ($kg\ ha^{-1}$)**	0.7	0.7	0.3	0.6	0.5	0.7	0.1	0.1	0.1

Table S2. APSIM soil and crop parameters used for the Innisfail site.

Depth (m)	0.0-0.30	0.30-0.60	0.60-1.00	1.00-1.50	1.50-1.80
**Organic C (%)**	2.09	1.03	0.74	0.46	0.23
**pH (water)**	5.16	4.81	4.58	4.75	4.98
**FBIOM (0 -1)**	0.030	0.015	0.015	0.010	0.010
**FINERT (0 - 1)**	0.40	0.60	0.90	0.99	0.99
**Bulk density ($g\ cm^{-3}$)**	1.19	1.09	1.24	1.31	1.35
**LL15 & sugar LL ($mm\ mm^{-1}$)**	0.21	0.14	0.21	0.25	0.29
**DUL ($mm\ mm^{-1}$)**	0.46	0.48	0.34	0.48	0.48
**SAT ($mm\ mm^{-1}$)**	0.55	0.59	0.53	0.50	0.49
**Sugar KL**	0.10	0.08	0.06	0.04	0.02
**Sugar XF**	1.0	1.0	1.0	1.0	1.0
**SWCON**	0.5	0.5	0.5	0.5	0.5
**NO_3-N ($kg\ ha^{-1}$)**	6.0	6.0	6.0	2.0	2.0
**NH_4-N ($kg\ ha^{-1}$)**	6.0	6.0	6.0	2.0	2.0

Table S3. APSIM soil and crop parameters used for the Mulgrave site.

Depth (m)	0-0.30	0.30-0.60	0.60-1.00	1.00-1.50	1.50-1.80
**Organic C (%)**	1.19	0.65	0.26	0.13	0.11
**pH (water)**	5.32	5.19	5.48	5.67	5.57
**FBIOM**	0.035	0.015	0.015	0.010	0.010
**FINERT**	0.40	0.60	0.99	0.99	0.99
**Bulk density (g cm^{-3})**	1.37	1.47	1.50	1.51	1.62
**LL15 & sugar LL (mm mm^{-1})**	0.05	0.18	0.17	0.06	0.04
**DUL (mm mm^{-1})**	0.24	0.40	0.38	0.26	0.20
**SAT (mm mm^{-1})**	0.48	0.45	0.43	0.43	0.39
**Sugar KL**	0.10	0.08	0.06	0.04	0.02
**Sugar XF**	1.0	1.0	1.0	0.0	0.0
**SWCON**	0.5	0.5	0.5	0.5	0.5
**NO_3-N (kg ha^{-1})**	9.7	5.3	4.5	4.5	4.2
**NH_4-N (kg ha^{-1})**	8.2	5.8	8.2	5.4	1.7

Table S4. APSIM soil and crop parameters used for the Maryborough site.

Depth (m)	0-0.10	0.10-0.20	0.20-0.40	0.40-0.60	0.60-0.90	0.90-1.20	1.20-1.50	1.50-2.00
**Organic C (%)**	1.12	1.12	0.70	0.36	0.25	0.23	0.22	0.22
**pH (water)**	5.48	5.48	5.75	6.03	5.59	5.24	4.81	4.81
**FBIOM**	0.035	0.020	0.015	0.015	0.015	0.010	0.010	0.010
**FINERT**	0.40	0.60	0.80	0.90	0.99	0.99	0.99	0.99
**Bulk density (g cm^{-3})**	1.71	1.71	1.59	1.48	1.42	1.59	1.54	1.62
**LL15 & sugar LL (mm mm^{-1})**	0.140	0.140	0.186	0.221	0.268	0.246	0.267	0.290
**DUL (mm mm^{-1})**	0.204	0.204	0.251	0.331	0.444	0.365	0.410	0.380
**SAT (mm mm^{-1})**	0.360	0.360	0.400	0.440	0.460	0.400	0.420	0.390
**Sugar KL**	0.10	0.10	0.08	0.06	0.04	0.02	0.01	0.01
**Sugar XF**	1.0	1.0	1.0	1.0	1.0	1.0	1.0	1.0
**SWCON**	0.5	0.5	0.5	0.4	0.4	0.4	0.4	0.4
**NO_3-N (kg ha^{-1})**	2.565	2.565	3.180	1.480	4.686	26.235	58.212	111.780
**NH_4-N (kg ha^{-1})**	2.052	2.052	2.957	2.072	4.260	7.632	2.772	4.050

Table S5. APSIM soil and crop parameters used for the Mossman site.

Depth (m)	0-0.20	0.20-0.40	0.40-0.70	0.70-1.00
**Organic C (%)**	1.23	0.74	0.39	0.27
**pH (water)**	5.71	5.61	5.66	5.64
**FBIOM**	0.030	0.020	0.015	0.010
**FINERT**	0.40	0.80	0.99	0.99
**Bulk density (g cm^{-3})**	1.31	1.36	1.53	1.38
**LL15 & sugar LL (mm mm^{-1})**	0.17	0.19	0.17	0.18
**DUL (mm mm^{-1})**	0.25	0.26	0.25	0.26
**SAT (mm mm^{-1})**	0.51	0.49	0.42	0.48
**Sugar KL**	0.08	0.06	0.02	0.01
**Sugar XF**	1.0	1.0	1.0	1.0
**SWCON**	0.65	0.65	0.65	0.65
**NO_3-N (kg ha^{-1})**	2.0	2.0	1.0	1.0
**NH_4-N (kg ha^{-1})**	1.0	1.0	1.0	1.0

Table S6. The meteorological station and APSIM soil water and crop parameters used for the Bundaberg, Mossman, Maryborough, Mulgrave, and Innisfail sites.

Parameter	Bundaberg	Mossman	Maryborough	Mulgrave	Innisfail
**Meteorological station**	Burnett heads	Mossman central mill	Maryborough	Gordonvale post office	South Johnstone Exp Stn
**Curve number (0-100)**	88	70	95	78	78
**Soil C:N ratio**	12.6	13.4	18.9	10.3	15.5
**Initial maximum available water (%)**	100	50	50	100	50
**Initial plant available water relative to LL15 (mm)**	139	39	112	355	201
**Rooting depth (m)**	1.75	1.0	2.0	1.0	1.8

Table S7. Non-default APSIM parameters used for simulating sugarcane production systems for the Bundaberg, Mossman, Maryborough, Mulgrave, and Innisfail sites.

APSIM module	Parameter name	Value	Site
**SoilN**	dnit_rate_coeff	0.001379	Innisfail; Mulgrave; Maryborough; Mossman; Bundaberg
**SoilN**	dnit_k1	25.1	Innisfail; Mulgrave; Maryborough; Mossman; Bundaberg
**SoilN**	nit_n2o_frac	0.002	Innisfail; Mulgrave; Maryborough; Mossman
**Sugar**	oxdef_photo_rtfr	0.5, 1.0	Innisfail
**Sugar**	oxdef_photo	1.0, 0.5	Innisfail
**Sugar**	oxdef_photo_rtfr	0.5, 0.8	Mulgrave; Maryborough; Mossman; Bundaberg
**Sugar**	oxdef_photo	1.0, 1.0	Mulgrave; Maryborough; Mossman; Bundaberg
**Sugar**	transp_eff_cf	0.0088	Bundaberg
**Sugar**	sen_detach_frac	1.0, 0.004 ,0.0 ,0.004 0	Bundaberg
**Sugar**	y_n_conc_crit_cane	0.0090, 0.0090, 0.0090, 0.0015 or 0.0025, 0.0015 or 0.0025	Bundaberg
**Sugar**	sen_detach_frac	1.0, 0.004 0.0, 0.004, 0	Bundaberg
**Surface Organic Matter**	crit_residue_wt	10000	Innisfail; Mulgrave; Maryborough; Mossman
**Surface Organic Matter**	opt_temp	30	Innisfail; Mulgrave; Maryborough; Mossman
**Surface Organic Matter**	pot_decomp (sugar)	0.06	Innisfail; Mulgrave; Maryborough; Mossman
**Surface Organic Matter**	pot_decomp (sugar)	0.025	Bundaberg

2 Regression tree analysis of factors affecting NUE

Regression tree analysis was performed using NUE as the continuous variable and a range of management, crop class (plant or ratoon) and site-specific factors as predictors. The management factors were N fertilizer rate, timing of N fertilizer application, splitting N applications in plant crops, fallow management, tillage, and in-field traffic management. The site specific factors were region, climate and soil type. Regression tree analysis was performed using the 'Rpart' package (Therneau et al., 2015) within the R statistical software (R Core Team, 2017). The criterion for splitting was the ‘anova method’ where the choice of split is done to maximize the between-groups sum of squares similar to a simple analysis variance. After ‘growing’ the initial full tree, ‘pruning’ was applied to avoid over-fitting the model. This was achieved by selecting the tree size (or number of splits) that

had a cross-validation error (x-error) greater than the minimum x-error plus one standard error. The proportion of variance explained by the pruned and full tree was calculated (pseudo-R^2 = 1 – relative error for the final split). A numerical indicator of the improvement in prediction for each was calculated during the analysis and the variable with the largest improvement in predictive accuracy was chosen as the *primary* predictor. So for each split, along with the *primary* predictors, there is ranking of *surrogate* predictors. This measure of predictive accuracy for *all* predictors was accumulated over the whole tree and used to rank the most influential predictors. Therefore it is possible for the *primary* predictor (i.e. the top split) to not be the most influential predictor across the whole tree.

3 Fitting empirical models to the simulated yield-N response functions

A continuous yield-N response function was required to calculate the economic optimum N fertilizer rate, and this continuous function was obtained by fitting empirical models to the simulated yield-N response functions. Given the large number of simulated functions we adopted an approach that allowed us to automate the fitting of empirical models. This approach consisted of fitting one of three models to the simulated yield-N response functions. These models were:

the Weibull model,

$$\boldsymbol{Yield = A + B \times e^{-e^{C} fert^{D}}} \quad \text{(S1)}$$

a four-parameter Logistic model,

$$\boldsymbol{Yield = E + \frac{F-E}{1+e^{(F-fert)/G}}} \quad \text{(S2)}$$

or a simple logistics model,

$$\boldsymbol{Yield = \frac{H}{1+e^{(I-fert)/J}}} \quad \text{(S3)}$$

where *Yield* is sugarcane yield (Mg ha^{-1}), *fert* is the N fertilizer rate (kg ha^{-1}) and *A, B, C, D, E, F, G, H, I,* and *J* are empirical constants determined in fitting the curves. The models were fitted using self-starting non-linear models found in the R-statistical programming software (R Core Team, 2017). Attempting to fit multiple models was important as the simulated yield-N response function shapes varied between the ‘classic’ curves with a ‘plateau’ at high N rates, to ones that had no plateau at higher N rates or were linear (as described in Section 3.2.1). Priority was given to fitting Weibull model, followed by four-parameter logistics and then the simple logistic model, with the procedure stopped when a good fit had been achieved. This order was selected from preliminary analysis of the simulated curves that indicated most had a shape that could be fitted to the Weibull model, followed by the other two models. To provide the continuous yield-N response function, the best model was used to predict the yield for N fertilizer rates from 25 to 300 kg ha^{-1} in 1 kg increments.

4 Calculation of partial gross margins

Partial gross margins (PGM) were determined from the continuous yield-N response functions described in Supplementary Material 1:

$$\boldsymbol{PGM} = \boldsymbol{Yield} * (\boldsymbol{Py} - \boldsymbol{HC}) - (\boldsymbol{fert} * \boldsymbol{Pn}) \qquad \text{(S4)}$$

where Py is the cane price (AU$30.14 Mg^{-1}), HC is the harvest costs (AU$7.73 Mg^{-1}), and Pn is the N fertilizer price (AU$1.38 kg N^{-1}).

5 References

Holzworth, D. P., Huth, N. I., deVoil, P. G., Zurcher, E. J., Herrmann, N. I., McLean, G., et al. (2014). APSIM – Evolution towards a new generation of agricultural systems simulation. *Environ. Model. Softw.* 62, 327–350. doi:10.1016/j.envsoft.2014.07.009.

Keating, B. A., Robertson, M. J., Muchow, R. C., and Huth, N. I. (1999). Modelling sugarcane production systems I. Development and performance of the sugarcane module. *F. Crop. Res.* 61, 253–271. doi:10.1016/S0378-4290(98)00167-1.

Muchow, R. C., and Robertson, M. J. (1994). Relating crop nitrogen uptake to sugarcane yield. *Proc. Aust. Soc. Sugar Cane Technol.* 122–130.

Probert, M. E., Dimes, J. P., Keating, B. A., Dalal, R. C., and Strong, W. M. (1998). APSIM's water and nitrogen modules and simulation of the dynamics of water and nitrogen in fallow systems. *Agric. Syst.* 56, 1–28. doi:10.1016/S0308-521X(97)00028-0.

R Core Team (2017). R: A language and environment for statistical computing. R Foundation for Statistical Computing, Vienna, Austria. URL https://www.R-project.org/.

Therneau, T., Atkinson, B., and Ripley, B. (2013). *Recursive Partitioning R package version 4.1-3*. Available at: http://CRAN.R-project.org/package=rpart.
